# Dynamic Encoding of Natural Luminance Sequences by LGN Bursts

**DOI:** 10.1371/journal.pbio.0040209

**Published:** 2006-06-13

**Authors:** Nicholas A Lesica, Chong Weng, Jianzhong Jin, Chun-I Yeh, Jose-Manuel Alonso, Garrett B Stanley

**Affiliations:** **1**Division of Engineering and Applied Sciences, Harvard University, Cambridge, Massachusetts, United States of America; **2**Department of Biological Sciences, State University of New York College of Optometry, New York, New York, United States of America; **3**Department of Psychology, University of Connecticut, Storrs, Connecticut, United States of America; University of California BerkeleyUnited States of America

## Abstract

In the lateral geniculate nucleus (LGN) of the thalamus, visual stimulation produces two distinct types of responses known as tonic and burst. Due to the dynamics of the T-type Ca ^2+^ channels involved in burst generation, the type of response evoked by a particular stimulus depends on the resting membrane potential, which is controlled by a network of modulatory connections from other brain areas. In this study, we use simulated responses to natural scene movies to describe how modulatory and stimulus-driven changes in LGN membrane potential interact to determine the luminance sequences that trigger burst responses. We find that at low resting potentials, when the T channels are de-inactivated and bursts are relatively frequent, an excitatory stimulus transient alone is sufficient to evoke a burst. However, to evoke a burst at high resting potentials, when the T channels are inactivated and bursts are relatively rare, prolonged inhibitory stimulation followed by an excitatory transient is required. We also observe evidence of these effects in vivo, where analysis of experimental recordings demonstrates that the luminance sequences that trigger bursts can vary dramatically with the overall burst percentage of the response. To characterize the functional consequences of the effects of resting potential on burst generation, we simulate LGN responses to different luminance sequences at a range of resting potentials with and without a mechanism for generating bursts. Using analysis based on signal detection theory, we show that bursts enhance detection of specific luminance sequences, ranging from the onset of excitatory sequences at low resting potentials to the offset of inhibitory sequences at high resting potentials. These results suggest a dynamic role for burst responses during visual processing that may change according to behavioral state.

## Abstract 2

This visual neuroscience paper simulates how resting potential and stimulus driven modulations in membrane potential interact to determine the response mode of LGN neurons to natural images.

## Introduction

Neurons in the lateral geniculate nucleus (LGN) of the thalamus exhibit two distinct types of responses known as tonic and burst (for review, see [ 1]). Bursts were originally observed during sleep and were thought to represent a decoupling of the LGN from its retinal input [ 2, 3]. However, recent studies have shown that bursts are interspersed with tonic firing in the LGN of awake animals during visual stimulation [ 4– 6] and that bursts can reliably encode visual information [ 7, 8]. Furthermore, other studies have shown that bursts are especially prevalent when the statistical properties of the visual stimulus resemble those of the natural world [ 9, 10].

LGN bursts are generated by low-threshold, voltage-dependent T-type Ca ^2+^ channels [ 11, 12]. When an LGN neuron is depolarized and responding in tonic mode, these channels are inactivated and have no effect on the response. However, if the neuron is hyperpolarized for a prolonged period of time, the channels are de-inactivated, and subsequent depolarization results in a slow calcium spike, which, in turn, causes a stereotyped burst of closely spaced action potentials. The relationship between the visual stimulus and the burst response is nonlinear, as weak stimuli are amplified to cause approximately the same response as strong stimuli [ 13]. Because of the low threshold and nonlinear amplification properties of burst responses, it is thought that LGN bursts may serve to enhance the detection of visual features [ 1]. Indeed, previous studies have shown that the ability of an LGN neuron to signal the appearance of visual features is increased when the neuron is in burst mode [ 14, 15], and also, importantly, that these effects cannot be reproduced simply by increasing the overall firing rate when the neuron is in tonic mode [ 16].

The LGN resting membrane potential is controlled by modulatory connections and varies according to behavioral state, reaching its lowest level during sleep and its highest level during active waking [ 17]. Because of the dynamics of the T-type Ca ^2+^ channels that control bursting, this modulatory control of resting potential can be an important factor in determining the LGN response to visual stimuli. In this study, we simulate LGN responses to natural scene movies using an integrate-and-fire-or-burst (IFB) model, which can accurately reproduce the LGN response during both tonic and burst firing [ 9, 18]. By analyzing responses to the same movie sequences at different resting potentials, we demonstrate that the interactions between modulatory and stimulus-driven changes in membrane potential determine the particular luminance sequences that evoke bursts. We also observe evidence of these interactions in vivo, where analysis of experimental recordings of cat LGN responses to the same movie sequences demonstrates that the luminance sequences that trigger bursts vary with the overall burst percentage (BP) of the response.

To investigate the functional consequences of the effects of resting potential on burst generation, we tested the effects of changes in resting potential on the extent to which bursts enhance the detection of different luminance sequences. Although comparing the LGN response with and without bursts by blocking T channels in vivo is not possible [ 19], these experiments can be simulated by disabling the burst mechanism in the IFB model. We simulated the LGN response to different temporal luminance sequences at a range of resting potentials with and without the burst mechanism. Analysis of these responses using signal detection theory shows that bursts enhance detection of different luminance sequences at different resting potentials. Taken together, the results of this study demonstrate that the interactions between modulatory and stimulus-driven changes in resting potential determine the luminance sequences that trigger LGN bursts and suggest that these interactions may have important functional implications.

## Results

### Simulated LGN Responses to Natural Scene Movies

**Figure 1:**
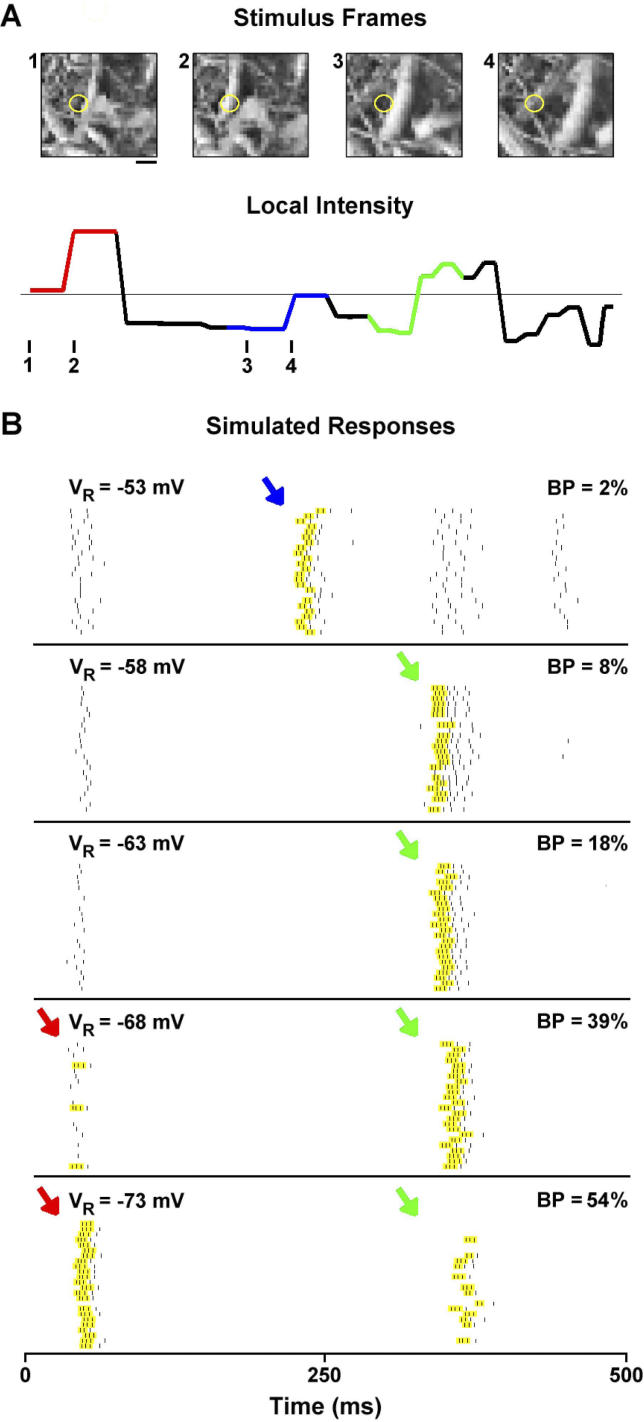
Simulated LGN Responses to Natural Scene Movies at Different Resting Potentials (A) Four frames of a natural scene movie are shown. Movies were recorded by a camera mounted on the head of a freely roaming cat [ 23]. The RF center of the simulated neuron is indicated by the yellow circle. The scale bar indicates 1°. The intensity of a 500-ms segment of the movie averaged over all pixels in the RF center is also shown. The thin gray line indicates the mean intensity. The tick marks on the intensity plot indicate the onset times of the corresponding frames. Three stimulus events are colored to correspond to burst events in the responses in (B). (B) Raster plots of simulated LGN responses to 24 repeats of the 500-ms segment of a natural scene movie shown in (A). Burst events in the responses are highlighted. The colored arrows next to each burst event correspond to the luminance sequences in (A). Responses were simulated using an IFB model. The resting potential of the model ( *V _R_*) and BP of the simulated response to a two-minute segment of natural scene movie that includes the 500-ms segment shown are indicated. The de-inactivation potential and threshold of the burst mechanism ( *V _T_*) was −60 mV. For values of other model parameters, see Materials and Methods.

The response of any sensory neuron depends on its resting potential. As the difference between the resting potential and the spike threshold changes, so will the timing and location of firing events in the response. These effects are especially strong in the LGN because, due to the dynamics of T channels, the resting potential determines the particular visual features that evoke a burst response. An illustration of these effects is shown in Figure 1. Figure 1A shows four frames from a natural scene movie, as well as the average intensity of the movie within the receptive field (RF) center (yellow circle) of a simulated neuron over a 500-ms segment. The tick marks on the intensity plot indicate the onset times of the corresponding frames. The mean luminance is shown by the thin gray line.

Figure 1B shows the simulated responses of an LGN neuron to the movie segment at different resting potentials (bursts are highlighted; for definition of bursts, see Materials and Methods). The responses were simulated using an IFB model. The model consists of a cascade of a spatiotemporal RF and an integrate-and-fire (IF) spike generation mechanism. Similar models have been used previously to describe the responses of X and Y cells in the cat LGN to white-noise and natural scene movie stimuli [ 9, 20]. The IFB model combines a low-threshold, voltage-dependent current with a traditional IF mechanism to mimic the dynamics of burst generation (for a full description of the model, see Materials and Methods).

It is clear from the results shown in Figure 1B that the LGN response, especially the occurrence of bursts, is strongly dependent on resting potential ( *V _R_*). In general, as *V _R_* increases, there is a decrease in the BP of the response (percentage of spikes involved in a burst, calculated over a long period of movie stimulation). At low resting potentials (bottom rows), when *V _R_* is much lower than the de-inactivation potential of the burst mechanism ( *V _T_* = −60 mV), burst events are evoked by the two luminance sequences (red and green) with excitatory transients that bring the local intensity above its mean value and, correspondingly, the membrane potential above its resting value. When the resting potential is increased so that *V _R_* is near *V _T_* (middle rows), only the biphasic sequence (green) evokes a burst, as inhibitory stimulation is needed to de-inactivate the burst mechanism and excitatory stimulation is needed to trigger the burst. When the resting potential is increased further so that *V _R_* is much higher than *V _T_* (top row), only the offset of the inhibitory sequence (blue) evokes a burst, as prolonged inhibitory stimulation is required to de-inactivate the burst mechanism and the return to rest evokes the post-inhibitory rebound (PIR) burst without the need for additional excitation.

### The Luminance Sequences That Evoke LGN Bursts

**Figure 2:**
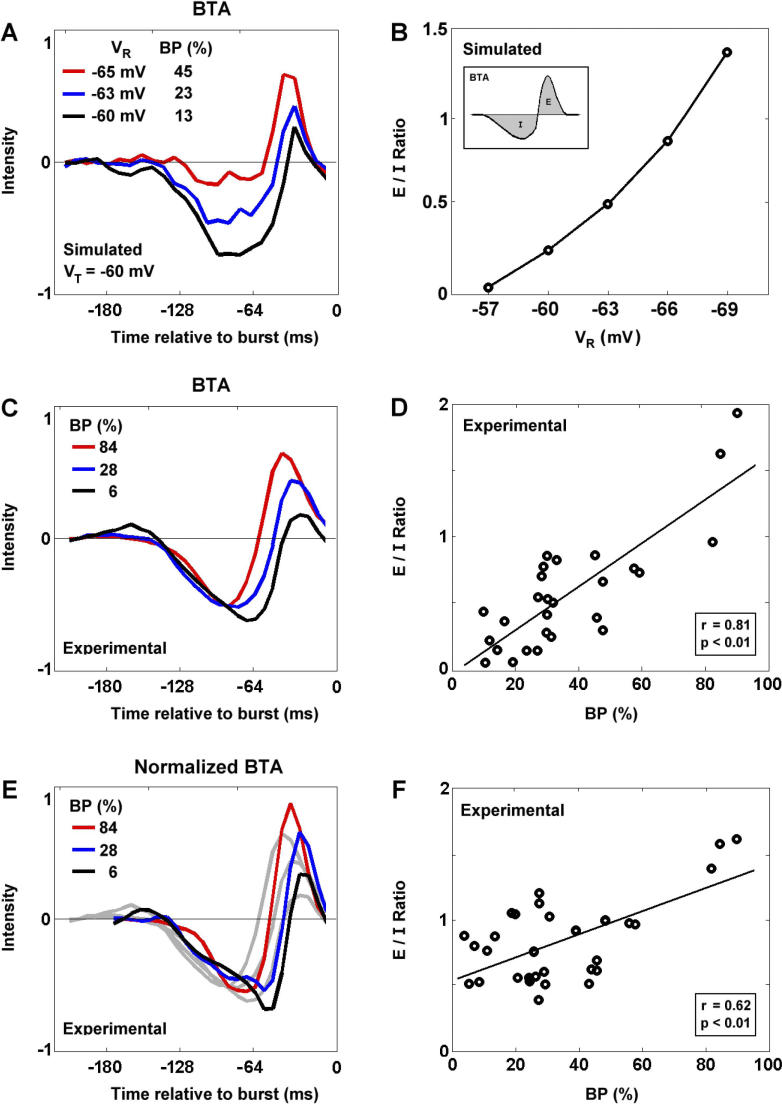
The Luminance Sequences That Trigger Burst Events (A) The BTAs calculated from simulated responses to a two-minute segment of natural scene movie at different resting potentials. The resting potential of the model and BP of the response corresponding to each BTA are indicated. Full spatiotemporal BTAs were calculated, and the plots show the intensity of the BTA averaged over all pixels in the RF center. Each BTA was scaled so that the integral of its absolute value was 1. (B) A plot of E/I ratio of the BTA versus resting potential for simulated responses to natural scene movies. E/I ratio was calculated as the ratio of the areas of the excitatory and inhibitory components of the BTA (see inset). (C) The BTAs calculated from the experimental responses of three LGN Y cells recorded at different times during a single experiment to natural scene movies (average of nine different two-minute segments). The BP of each response is indicated. Spatiotemporal BTAs were averaged and scaled as in (A). (D) A scatter plot of E/I ratio of the BTA versus BP for a sample of 27 LGN cells (11 X cells, 16 Y cells). E/I ratio was calculated as described in (B). (E) The normalized BTAs for three LGN Y cells. BTAs were normalized for the temporal correlations in the natural scene movies by spectral normalization (see Materials and Methods). The non-normalized BTAs corresponding to each normalized BTAs are shown in gray (same BTAs as in (C)). (F) A scatter plot of E/I ratio of the normalized BTA versus BP for a sample of 27 LGN cells. E/I ratio was calculated as described in (B).

To further investigate the effects of resting potential on the generation of bursts, we simulated the LGN response to natural scene movies at different resting potentials as described above and calculated the average stimulus that preceded a burst response, known as the burst-triggered average (BTA) (see Methods). The BTAs calculated from simulated responses at three different resting potentials are shown in Figure 2A. When *V _R_* = −65 mV, which is well below *V _T_* = −60 mV, the BP in the response is relatively high (45%) and BTA consists of an excitatory transient that brings the intensity above its mean value (red). At this low resting potential, the burst mechanism is de-inactivated at rest, so little inhibitory stimulation is needed and an excitatory stimulus alone can trigger a burst. When *V _R_* is increased to −63 mV, the BP decreases (23%) and the BTA becomes biphasic (blue). In this case, the resting potential is closer to *V _T_*, so inhibitory stimulation is needed to reliably de-inactivate the burst mechanism (because of the noise in the membrane potential) before excitatory stimulation can trigger a burst. Finally, when *V _R_* is increased to −60 mV, so that the burst mechanism is inactivated at rest, the BP is relatively low (13%) and the BTA consists of a prolonged inhibitory stimulus followed by an excitatory transient that brings the intensity back to its mean value (black). In this case, strong inhibitory stimulation is needed to de-inactivate the burst mechanism, and little excitatory stimulation beyond the return to rest is needed to trigger the PIR burst. These effects are summarized in Figure 2B, which shows that the ratio of the areas of the excitatory and inhibitory components of the BTAs (E/I ratio, see inset) increases as the resting potential decreases.

The BTAs in Figure 2A indicate that under all conditions, an excitatory stimulus transient is required to evoke a burst, while the position of the resting potential ( *V _R_*) relative to the burst de-inactivation potential ( *V _T_*) determines the strength of the inhibitory stimulus that must precede the excitatory transient. Evidence of this effect is also observable in experimentally recorded LGN responses to the same natural scene movies. While resting potential cannot be determined directly from extracellular recordings, studies using intracellular recordings both in vitro and in vivo have demonstrated a strong correlation between resting potential and BP [ 12, 21], and this relationship is also evident in our simulated responses (see Figure 1). Figure 2C shows the BTAs for three cells recorded at different times during a single experiment. The relationship between BP and BTA shape in the experimental responses mirrors that observed for resting potential and BTA shape in the simulated data above. This effect is summarized in Figure 2D which shows a strong correlation between BP and E/I ratio of the BTA ( *r* = 0.81, *p* < 0.01) for a sample of 27 LGN cells.

The BTAs described above reflect not only the selectivity of the burst response, but also the temporal frequency content of the natural scene movies. Because natural scene movies contain most of their power at low temporal frequencies [ 9, 22, 23], these frequencies are overrepresented in the BTA. By normalizing the BTAs for the temporal frequency content of the movies (see Materials and Methods), we can compensate for the overrepresentation of low frequencies and obtain an approximate characterization of the luminance sequence to which these neurons have the highest burst sensitivity. This normalization also facilitates comparison with previous studies of BTAs during white-noise stimulation (see Discussion). The normalized BTAs for the same three cells shown in Figure 2C are shown in Figure 2E. The corresponding non-normalized BTAs are shown in gray. Compared with the non-normalized BTAs, the normalized BTAs have a faster time course and are more biphasic. However, the relationship between BP and E/I ratio that was evident in the analysis of the non-normalized BTAs is also evident in the analysis of the normalized BTAs ( Figure 2F, *r* = 0.62, *p <* 0.01).

### A Simulated Detection Task

As LGN bursts have been implicated in the detection of visual features, the effects of resting potential on burst generation described above may have important functional consequences. To explore the effects of resting potential on the detection of visual features, we utilized the IFB model described above to simulate the LGN response and analyzed the results using signal detection theory. By disabling the burst mechanism in the IFB model, it can be reduced to a standard IF model, allowing the LGN response with and without bursts to be explicitly compared.

In a natural setting, the LGN must signal the appearance of visual features that are embedded in noise (for example, the activation of a vehicle's brake lights viewed through a rainy windshield). To simulate this situation, we presented a series of different temporal luminance sequences in combination with additive noise. Based on the different shapes of the BTAs shown in Figure 2A, we simulated the LGN response to three sequences: excitatory (corresponding to the BTA for the lowest resting potential, shown in red), inhibitory (corresponding to the BTA for the highest resting potential, shown in black), and biphasic (corresponding to the BTA for the middle resting potential, shown in blue). The dynamics of the sequences were chosen to match those of the BTAs (before normalizing for the correlations in the natural scene movies), as they represent the actual luminance sequences of the movies that triggered burst responses. The task we simulated is one in which a hypothetical observer must decide whether or not the luminance sequence of interest has appeared at each time interval based only on the LGN response during that interval. The intensity and appearance times of the sequences were chosen randomly and the intensity of the noise was varied relative to the intensity of the sequence to provide a range of stimulus signal-to-noise ratios (SNRs) (see Materials and Methods for details).

**Figure 3:**
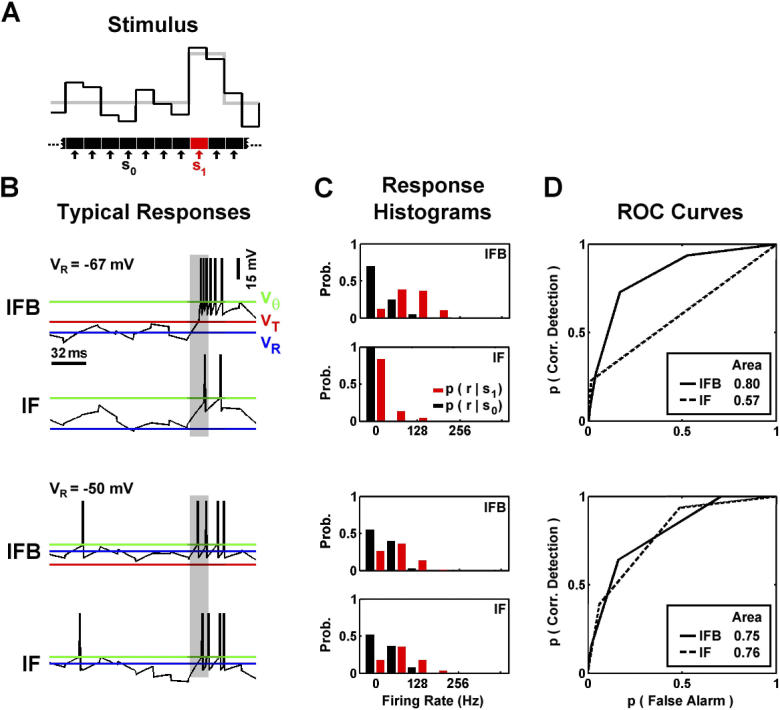
Detection of the Onset of Excitatory Luminance Sequences (A) LGN responses to a noisy stimulus in which an excitatory sequence randomly appeared were simulated with and without bursts using the IFB and IF models (see Materials and Methods). The stimulus was classified as *S*_0_ (black) or *S*_1_ (red), depending on whether or not each interval contained the excitatory transient of the sequence. A typical realization of the stimulus with SNR = 1/2 is shown (intensity averaged over all pixels in RF center). The black line indicates the actual stimulus and the gray line indicates the underlying sequence. (B) Voltage traces of the IFB and IF responses to the stimulus shown in (A) at two different resting potentials, *V _R_* = −67 mV (top) and *V _R_* = −50 mV (bottom), with *V _T_* = −60 mV. The interval in the response that corresponds to condition *S*_1_ is shaded. (The response was shifted for presentation to remove latency between stimulus and response). The spike threshold ( *V _Θ_,* green), burst de-inactivation potential and threshold ( *V _T_,* red), and resting potential ( *V _R_,* blue) are shown. (C) The probability distributions of the firing rate of the IFB and IF models during the *S*_0_ (black) and *S*_1_ (red) stimulus conditions at *V _R_* = −67 mV (top) and *V _R_* = −50 mV (bottom) with stimulus SNR = 1/2. Distributions were calculated using the response to a stimulus segment that contained 100 sequences. (D) ROC curves for the IFB and IF models at *V _R_* = −67 mV (top) and *V _R_* = −50 mV (bottom) calculated from the distributions in (C) using likelihood ratios as described in Materials and Methods. The area under the ROC curve is indicated.

A typical trial for the detection of the onset of an excitatory sequence (where the appearance of the sequence brings the intensity of the stimulus above its mean value) with stimulus SNR = 1/2 is shown in Figure 3A. The plot shows the intensity of the noisy stimulus (black), superimposed on the underlying sequence (gray). The temporal scale of the sequence was chosen to match the BTA for the lowest resting potential, shown in red in Figure 2A. Figure 3B shows the IFB and IF responses to the stimulus shown in Figure 3A. The top two plots show the IFB and IF responses when the resting potential of the model *V _R_* = −67 mV (with the burst de-inactivation potential and threshold *V _T_* = −60 mV and the action potential threshold *V _Θ_* = −45 mV). Because the burst mechanism is de-inactivated at rest, the IFB model responds to the onset of the excitatory sequence with a burst, while the IF model responds with tonic spiking. The bottom two plots show the IFB and IF responses when *V _R_* = −50 mV. Because the burst mechanism is now inactivated at rest, both models respond to the onset of the excitatory sequence with tonic spiking.

To effectively signal the appearance of a luminance sequence, the response of the neuron to the appearance of the sequence must be significantly different from the response in its absence. To compare the responses of the models (firing rate in 16-ms bins, denoted *r*) in the presence and absence of a sequence, the responses during intervals that contained the excitatory transient of a sequence (denoted *S*_1_) were separated from the responses during all other intervals (denoted *S*_0_, see Figure 3A) . The probability distributions of the IFB and IF responses under the *S*_1_ (red) and *S*_0_ (black) stimulus conditions (with stimulus SNR = 1/2) are shown in Figure 3C. Because of the noise in the stimulus, the response distributions under the two stimulus conditions overlap. However, when *V _R_* = −67 mV, the low threshold and nonlinear amplification properties of the burst mechanism allow the IFB model to produce a larger response to the onset of the excitatory sequence than that of the IF model. Because the effects of the burst mechanism are temporally localized (i.e., the responses to certain stimuli are amplified, but the responses to other stimuli are not affected), bursts help to separate the response distributions of the IFB model under the *S*_1_ and *S*_0_ conditions. Similar effects cannot be achieved by increasing the overall gain or firing rate of the IF model, as this would affect the response to noise and the response to sequences equivalently, and the separation between the response distributions would remain the same.

If the observer is to use the LGN response to decide whether or not a luminance sequence has appeared, the reliability of the decision will depend on the amount of overlap between the response distributions under the *S*_1_ and *S*_0_ and stimulus conditions. If the two response distributions are not overlapping, then the observer can easily draw a threshold to separate them. As the degree of overlap between the two distributions increases, part of one or both distributions may lie on the opposite side of the threshold such that some fraction of the responses will be classified incorrectly. The reliability of the observer's decision ( *D*_1_ = sequence appeared, *D*_0_ = no sequence appeared) can be quantified by calculating the area under the receiver operating characteristic (ROC) curves formed from the two response distributions (specifically, the area under the ROC curve is the probability of a correct decision in a two-alternative forced-choice task, see Materials and Methods for details). The ROC curves plot the probability of detection *p*( *D*_1_ | *S*_1_) versus the probability of false alarm p( *D*_1_ | *S*_0_) for all possible threshold values. The ROC curves for the IFB and IF models in the detection of the onset of the excitatory sequence are shown in Figure 3D. When *V _R_* = −67 mV, the low threshold and nonlinear amplification of the burst mechanism in the IFB model result in a much larger ROC area (solid line, 0.80) compared with that of the IF model (dashed line, 0.57). When *V _R_* = −50 mV, the ROC areas for the IFB and IF models are similar, as both respond to the appearance of a sequence with tonic firing. Thus, detection of the onset of the excitatory luminance sequence is enhanced at *V _R_* = −67 mV, when the burst mechanism is de-inactivated at rest, but not at *V _R_* = −50 mV, when the burst mechanism is inactivated at rest.

**Figure 4:**
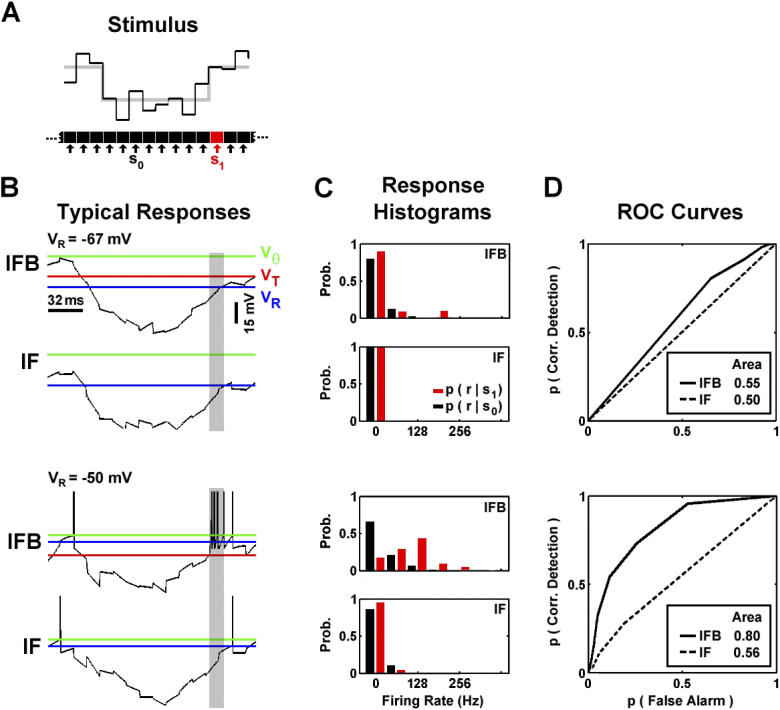
Detection of the Offset of Inhibitory Luminance Sequences (A) LGN responses to a noisy stimulus in which an inhibitory sequence randomly appeared were simulated. The stimulus was classified as *S*_0_ (black) or *S*_1_ (red) depending on whether or not each interval contained the excitatory transient of the sequence. A typical realization of the stimulus with SNR = 1/2 and sequence duration = 128 ms is shown (intensity averaged over all pixels in RF center). The black line indicates the actual stimulus and the gray line indicates the underlying sequence. (B) Voltage traces of the IFB and IF responses to the stimulus shown in (A) at two different resting potentials, *V _R_* = −67 mV (top) and *V _R_* = −50 mV (bottom), with *V _T_* = −60 mV. The interval in the response that corresponds to condition *S*_1_ is shaded (response was shifted for presentation to remove latency between stimulus and response). The spike threshold ( *V _Θ_,* green), burst de-inactivation potential and threshold ( *V _T_,* red), and resting potential ( *V _R_,* blue) are shown. (C) The probability distributions of the firing rate of the IFB and IF models during the *S*_0_ (black) and *S*_1_ (red) stimulus conditions at *V _R_* = −67 mV (top) and *V _R_* = −50 mV (bottom). (D) ROC curves for the IFB and IF models at *V _R_* = −67 mV (top) and *V _R_* = −50 mV (bottom). The area under the ROC curve is indicated.

We also tested the effects of resting potential on the detection of the offset of inhibitory luminance sequences (where the appearance of the sequence brings the intensity of the stimulus below its mean value). The task was identical to the previous one, except that the temporal profile of the sequence was matched to the BTA for the highest resting potential, shown in black in Figure 2A. A typical trial with stimulus SNR = 1/2 is shown in Figure 4A, along with the corresponding IFB and IF responses in Figure 4B. When *V _R_* = −67 mV, neither model responds to the offset of the inhibitory sequence. However, when *V _R_* = −50 mV, the hyperpolarization induced by the onset of the sequence de-inactivates the burst mechanism in the IFB model and the return to rest following the offset of the sequence triggers a PIR burst. Because the threshold for action potential generation is much higher than that for burst generation, the IF model remains silent.

The probability distributions of the IFB and IF responses under the *S*_1_ (red) and *S*_0_ (black) stimulus conditions (with stimulus SNR = 1/2) are shown in Figure 4C. Again, the *S*_1_ stimulus condition corresponds to those intervals that contain the excitatory transient of the luminance sequence, while the *S*_0_ stimulus condition corresponds to all other intervals (see Figure 4A). When *V _R_* = −67 mV, the response distributions of the two models are similar, with the response during both stimulus conditions typically zero (i.e., not spiking). This is reflected in the ROC curves shown in Figure 4D, and the performance of both models in the detection task is relatively poor. When *V _R_* = −50 mV, the IF response distributions under the two stimulus conditions remain similar, while the PIR bursts help to separate the IFB response distributions. This is reflected in the large ROC area (0.80) of the IFB model relative to that of the IF model (0.56). Thus, detection of the offset of the inhibitory sequence is enhanced at *V _R_* = −50 mV, because of PIR bursts, but not at *V _R_* = −67 mV.

**Figure 5:**
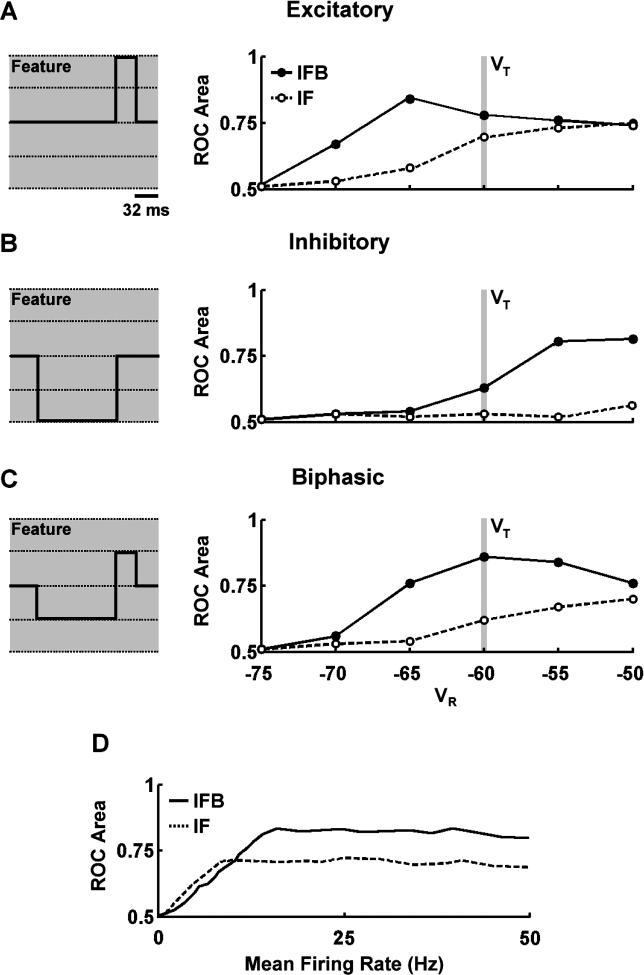
Sequence Detection at Different Resting Potentials (A) The temporal profile of the excitatory sequence and the area under the ROC curves for the IFB and IF models in the excitatory sequence detection task at different resting potentials with stimulus SNR = 1/2. (B) The temporal profile of the inhibitory sequence and the ROC areas for the IFB and IF models in the task involving the detection of the offset of inhibitory sequences at different resting potentials with stimulus SNR = 1/2. (C) The temporal profile of the biphasic sequence and the ROC areas for the IFB and IF models in the biphasic sequence detection task at different resting potentials with stimulus SNR = 1/2. (D) The ROC areas for the IFB and IF models in the biphasic sequence detection task at different overall firing rates with stimulus SNR = 1/2 and *V _T_* = −60 mV. The mean firing rate of the models was varied by changing the gain of the filter relating stimulus intensity to membrane potential.

Figures 3 and 4 show the results of detection simulations at two different resting potentials. The observed effects are summarized for a wide range of resting potentials in Figure 5. The data shown are for detection tasks with stimulus SNR = 1/2, but the trends illustrated were evident across the range of SNRs that we tested. Figure 5A shows the ROC areas for the IFB (solid) and IF (dashed) models in the detection of the onset of the excitatory sequence for resting potentials ranging from −75 mV to −50 mV. Detection of the onset of the excitatory sequence is greatly enhanced at low resting potentials, when the burst mechanism is de-inactivated at rest and the resting potential is far from the action potential threshold. As the resting potential increases, the difference in the ROC areas decreases, and when the burst mechanism is inactivated at rest ( *V _R_ > V _T_*), the ROC areas for the IFB and IF models are similar. Figure 5B shows the ROC areas for the detection of the offset of the inhibitory sequence across the same range of resting potentials. At low resting potentials, the ROC areas for the IFB and IF models are similar, as the offset of the inhibitory sequence generally fails to evoke a response in either model, while at high resting potentials, PIR bursts enhance the detection of the offset of the inhibitory sequence.

Figure 5C shows the ROC areas for a third detection task in which a biphasic sequence was presented. The temporal scale of the biphasic sequence was matched to the BTA for the middle resting potential, shown in blue in Figure 2A, and the amplitude of the excitatory and inhibitory phases of the sequence were set to half that of the corresponding monophasic sequences shown in Figure 5A and 5B. In this task, detection is enhanced by the burst mechanism at resting potentials near *V _T_,* as indicated by the difference in the ROC areas for the IFB (solid) and IF (dashed) models. The hyperpolarization induced by the inhibitory phase of the biphasic sequence will de-inactivate the burst mechanism if it is resting above *V _T_* and have little effect otherwise, and the excitatory phase of the biphasic sequence will evoke the burst from resting potentials below *V _T_,* when the return to rest after the offset of the inhibitory phase of the sequence alone is not enough to evoke a PIR burst.

The results shown in Figure 5 suggest that changes in the LGN resting potential can have significant functional consequences. By modulating the likelihood that a particular luminance sequence will evoke a burst, changes in resting potential also affect the degree to which the detection of different sequences is enhanced by the burst mechanism. At resting potentials well below *V _T_,* the strongest enhancement is in the detection of the onset of purely excitatory sequences. However, as the resting potential is increased toward *V _T_,* the enhanced detection of excitatory sequences decreases and the detection of the excitatory transient of biphasic sequences is strongly enhanced. Finally, as the resting potential is increased above *V _T_,* the enhanced detection of biphasic sequences decreases and the strongest enhancement is in the detection of the offset of purely inhibitory sequences.

It is important to note that the enhancement in detection provided by the burst mechanism is not due simply to an increase in overall firing rate. The burst mechanism enhances detection by increasing the response to the appearance of the sequence without increasing the response to noise. Thus, the same effect could not be achieved simply by increasing the gain (and, thus, the overall firing rate) of the IF model, as the response to the appearance of the sequence and the response to noise would both be increased. This is illustrated in Figure 5D, which shows the ROC area for the IFB and IF models in the biphasic sequence detection task ( *V _T_* = −60 mV) as a function of overall firing rate. When the gain (and firing rate) of the models is low, not all appearances of the sequence evoke a response and increasing the gain of the models can increase the ROC area. However, once the gain of the models is high enough that all appearances of the sequence evoke a response, further increases do not increase the ROC area. Because of the nonlinear amplification properties of the burst mechanism, the IFB model is able to signal the appearance of the sequence more reliably than the IF model, even when the IF model has a much higher overall firing rate.

## Discussion

The dynamics of T channels dictate that LGN bursts will occur when the membrane potential is below the de-inactivation and threshold potential of the channels ( *V _T_*) for a prolonged period of time and then rises above it. To characterize how modulatory and stimulus-driven changes in membrane potential interact to determine the luminance sequences that evoke bursts, we analyzed simulated LGN responses to natural scene movies. Our analysis demonstrates that an excitatory stimulus transient is required to evoke a burst at all resting potentials. When the resting potential is low ( *V _R_* < *V _T_*), and the burst mechanism is de-inactivated at rest, an excitatory transient alone can trigger a burst. However, at high resting potentials ( *V _R_* ≥ *V _T_*), when the T channels are inactivated at rest, a prolonged inhibitory stimulus will be required to de-inactivate the channels before an excitatory stimulus can evoke a burst. Furthermore, if the resting potential is above the threshold at which the T channels open ( *V _R_* ≥ *V _T_*), the depolarization that results from the offset of the inhibitory stimulus alone may be enough to evoke a PIR burst, with no additional excitation necessary.

These results are evident in the BTAs calculated from simulated LGN responses to natural scene movies at different resting potentials shown in Figure 2A. At all resting potentials, the BTA contains a strong excitatory transient that peaks 30–50 ms before the burst. At low resting potentials, the inhibitory stimulus preceding the excitatory transient is relatively small, and the BTA resembles the onset and offset of an excitatory luminance sequence. As the resting potential increases, the size of the inhibitory stimulus that precedes the excitatory transient in the BTA increases, such that at high resting potentials the BTA resembles the onset and offset of an inhibitory luminance sequence. Several other studies have characterized the stimulus features that trigger LGN bursts from responses to white noise as biphasic in time, consisting of a prolonged inhibitory phase followed by an excitatory transient [ 7– 9]. However, in describing the stimulus features that triggered bursts, these studies characterized bursting as a static encoding mechanism and ignored the effects of the interactions between modulatory and stimulus-driven changes in membrane potential that are evident in our simulated results. Here, a more detailed analysis of in vivo responses similar to those recorded in previous studies shows evidence of these interactions, as the luminance sequences that evoke bursts (BTAs) vary dramatically with overall BP ( Figure 2C– 2D). Note that the non-normalized BTAs in our study ( Figure 2C– 2D) have slower temporal dynamics than those reported previously, as they reflect the strong temporal correlations in the natural scene movies. The timescales of the BTAs that have been normalized for the correlations in the natural stimuli ( Figure 2E– 2F) are comparable to those described in previous studies [ 7– 9] and exhibit a dependence on BP similar to that observed for the non-normalized BTAs.

The effects of resting potential on the generation of bursts can have a significant impact on the detection of luminance sequences. The low threshold and nonlinear amplification properties of the burst mechanism can result in a large response to specific luminance sequences when the response in the absence of the burst mechanism would otherwise be small or nonexistent. We used the IFB and IF models to simulate the LGN response to the appearance of temporal luminance sequences with and without the burst mechanism, and compared the results using signal detection theory. The results shown in Figures 3– 5 demonstrate that the detection of the onset of excitatory stimulus sequences (sequences that bring the intensity of the stimulus above its mean value) is enhanced at low resting potentials, while the detection of the offset of inhibitory luminance sequences (sequences that bring the intensity of the stimulus below its mean value) is enhanced at high resting potentials. Furthermore, we demonstrate that the observed enhancement in detection is a result of the temporally localized increase in gain provided by the burst response, as similar enhancement in detection cannot be achieved simply by increasing the overall gain (or firing rate) of a neuron in tonic mode ( Figure 5D). The results we have presented in the context of natural luminance sequences are consistent with other studies of detection in the LGN that have demonstrated the ability of bursts to enhance the detection of sinusoidal gratings and spots [ 14, 15] and excitatory and inhibitory current inputs [ 16]. It should also be noted that, while we focused on the temporal dynamics of luminance sequences, the natural environment also exhibits strong spatial correlations. These correlations are important for evoking strong stimulus-driven changes in membrane potential (given linear integration of the stimulus across the spatial extent of the LGN RF), and spatial uniformity of the features in our detection task was explicitly assumed.

Our analysis of the detection simulations includes two important assumptions: 1) the relevant information in LGN response is carried in the firing rate over short time intervals, specifically in the instantaneous firing rate in 16-ms bins; and 2) downstream neurons have some knowledge of the probability distributions of the LGN response in the presence and absence of the sequence, *p*( *r* = *R* | *S*_1_) and *p*( *r* = *R* | *S*_0_), and respond differently under each condition. In fact, these assumptions are related, and their justification lies in the integration of LGN inputs at the thalamocortical synapse. Studies have reported that there is temporal summation of homosynaptic thalamic inputs to the cortex that arrive within a 16-ms window, with the arrival of a second input more than doubling the probability of evoking a cortical spike and the arrival of a burst often evoking multiple cortical spikes [ 24, 25]. Since the LGN response in the absence of a stimulus is typically zero or one spike, and the burst response to the appearance of a sequence is typically many spikes, the appearance of the sequence is likely to evoke a cortical response, while its absence is significantly less likely. Thus, the thalamocortical synapse appears to implement a decision criterion that is consistent with our assumptions.

There are a number of other properties of the LGN response that may be important for detection that our analysis did not consider. For example, it has been shown that the silence that precedes an LGN burst is important in evoking a cortical response, as it allows the synapse to recover from any depression induced by recent activity before the burst arrives [ 25]. Furthermore, while experiments have shown that a vast majority of the visual information in the response of an LGN cell is contained in the firing rate, there appears to be significant additional information in spike timing [ 26], and cortical neurons possess the necessary machinery to decode this additional information [ 27]. While burst responses are highly stereotyped and there is little evidence of visual information in the precise timing of spikes within an individual burst [ 28], the stereotyped nature of the burst response could be exploited to distinguish burst responses from other high frequency firing events. It is likely that further analysis of the role of LGN bursts in the detection of stimulus features that consider the history dependence or stereotyped nature of the burst response would reveal even stronger effects than the analysis of time-varying firing rate presented here.

Several authors have hypothesized a role for the thalamus in the direction of attention [ 29, 30] and these hypotheses have been confirmed by lesion studies in humans and rats [ 31, 32]. Specific roles for LGN bursts in bottom-up or involuntary attention have also been proposed. One hypothesis suggests that bursts may serve as a “wake-up call,” alerting the cortex to the presence of a salient stimulus [ 33], while another proposes that bursts may be important in low-level control of visual information [ 34]. Our results support these hypotheses and demonstrate that bursts could be used as a reliable signal to direct the deployment of attentional resources to a behaviorally relevant area of the visual field. Further evidence linking LGN bursts and attention was provided by a recent experimental study that examined bursting in awake rabbits during attentive and inattentive states [ 35]. The study reports significant changes in BP and rapid shifts in visual feature selectivity following transitions between attentive and inattentive states.

Considering our results along with previous studies of thalamic bursts, we envision a typical behavioral scenario as follows: assuming the animal is awake but passive, brainstem activity sets the LGN baseline membrane potential just below *V _T_,* such that the T channels are de-inactivated at rest, and the appearance of an excitatory stimulus will evoke a burst [ 36, 37]. When a salient stimulus appears, a burst is triggered, evoking a strong cortical response, raising the animal's level of alertness, and capturing its attention [ 25]. The combination of stimulus-driven and attentionally modulated cortical feedback, as well as increased brainstem activity due to increased arousal, depolarizes the cell so that its resting potential rises above *V _T_,* increasing the spontaneous firing rate for detailed transmission of both excitatory and inhibitory stimulus features via tonic firing [ 13, 38, 39]. Our results suggest that while the stimulus retains the animal's attention and the cell remains depolarized, the burst mechanism would only be activated by prolonged inhibitory stimuli. These PIR bursts may signal that the current level of depolarization is insufficient to transmit the inhibitory modulations in the stimulus, and the strong cortical response evoked by the burst may increase feedback and further depolarize the cell. When the stimulus disappears (or its salience level is sufficiently decreased) and the animal returns to the passive state, the cycle repeats. To determine whether LGN bursts do indeed serve such a dynamic role during natural vision, further study of visual processing in awake animals during different behavioral states is required.

## Materials and Methods

### Natural scene movie stimuli.

Movie sequences were recorded by members of the laboratory of Peter König (Institute of Neuroinformatics, ETH/UNI Zürich, Switzerland) using a removable lightweight CCD-camera mounted to the head of a freely roaming cat in natural environments such as grassland and forest [ 23]. Example movies are shown in Videos S1– S3. Movies were recorded via a cable connected to the leash onto a standard VHS-VCR (Pal) carried by the human experimenter and digitized at a temporal resolution of 25 Hz. Each frame of the movies consisted of 320 × 240 pixels and 16 bit color depth. For this study, the movies were converted to 8-bit gray scale and a 64 × 64 section of each frame was used. To improve temporal resolution, movies were interpolated by a factor of two (to a sampling rate of 50 Hz) using commercial software (MotionPerfect, Dynapel Systems, New York, New York, United States). Following interpolation, the intensities of each movie frame were rescaled to have a mean value of 125 (possible values were 0–255) and an RMS contrast of 0.45. During experimental presentation, movies were shown on a 20-in monitor with a refresh rate of 120 Hz, with pixel intensities updated every other refresh so that playback approximated the intended temporal resolution of the interpolated movies. The spatial resolution of the stimulus was such that each pixel was a square measuring 0.2° per side. For each cell, nine different two-minute segments of movie were shown, along with 24 repeated trials of a different 90s segment of movie. These stimuli were also used to simulate the LGN response using the IF and IFB models as described below.

### Definition of burst events.

Bursts were defined according to the standard criterion [ 7, 9, 12]. A burst was a group of two or more action potentials, each of which is less than 4 ms apart, with the first spike preceded by more than 100 ms of silence. Intracellular studies have shown that this criterion is effective for selecting only those spikes originating from calcium-induced bursts [ 12]. Burst identification was based on spike times at 0.1-ms resolution.

### Simulation of LGN responses.

We simulated LGN responses to visual stimuli using an IFB model [ 9, 18]. The IFB model consists of a cascade of a RF and a spike generation mechanism, and is similar to those used to describe the responses of X and Y cells in the cat LGN to white-noise and natural scene movie stimuli [ 9, 20]. In the model, the spatiotemporal stimulus is summed over space and time using a model RF with center/surround spatial profile and biphasic temporal profile typical of LGN cells [ 40]. The spatial profile is defined by the radially symmetric difference of Gaussians:



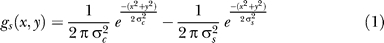



and the temporal dynamics are defined by the biphasic function:



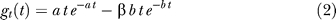



with delay *δ*. To model the spatiotemporal inseparability of LGN RFs, we used two separate temporal functions, 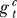 and 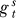, to modulate the center and surround Gaussians independently. All simulated responses were generated using the following model RF parameters: *β _c_* = 1, *β _s_* = 1, *a _c_* = 1/0.01 s, *a _s_* = 1/0.012 s, *b _c_* = 1/0.011 s, *b _s_* = 1/0.013 s, *σ _c_* = 0.5°, *σ _s_* = 0.65°, *δ _c_* = 24 ms, and *δ _s_* = 32 ms. These parameters were chosen so that the spatiotemporal integration properties of the model RF were similar to those of typical cat LGN cells [ 41]. The model RF was scaled such that the integral of its absolute value was one.

The visual stimulus is convolved with the RF, scaled by a constant value *α* and used as input to a stochastic IFB spike generator. The response of the IFB spike generator is determined by the following system of equations:



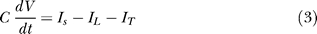





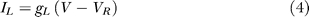





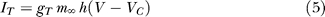





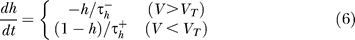



where *C* is the membrane capacitance, *V* is the membrane potential, *I _s_* is the input current defined by the filtered and scaled stimulus, *I _L_* is the leakage current (assuming constant conductance), *V _R_* is the resting potential, *I _T_* is the burst-related calcium current, and *V _C_* is the calcium reversal potential. The activation of the calcium current is controlled by *m*_∞_ = *H*( *V*− *V _T_*), where *H* is the Heaviside step function and *V _T_* is the membrane potential below which the burst mechanism becomes de-inactivated. The dynamics of the calcium current are controlled by 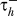, which sets the duration of hyperpolarization necessary to de-inactivate the burst mechanism, and by 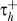, which sets the duration of the calcium spike. When the membrane potential crossed the threshold value *V _Θ_,* a spike was generated and the membrane potential was set to *V _RESET_* on the next time step. The model was made stochastic by adding Gaussian noise with zero mean and a standard deviation of *σ _n_* mV to the membrane potential. All simulated responses were generated using the following parameters: *α* = 3, *C* = 2 *μ* F/cm ^2^, *g _L_* = 0.035 mS/cm ^2^, *V _RESET_* = −50 mV, *V _Θ_* = −45 mV, *V _T_* = −60 mV, *V _C_* = 120 mV, 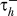 = 20 ms, 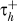 = 100 ms, *g _T_* = 0.07 mS/cm ^2^, and *σ _n_* = 1 mV. These parameters were chosen to match those that have been used to describe the responses of cat LGN cells to current inputs and natural scene movie stimulation [ 9, 16, 18].

To simulate the LGN response with bursts, the IFB model was used as described above. To simulate the LGN response without bursts using a standard IF model, the current 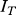 was set to zero.

### Recordings from cat LGN.

The surgical and experimental preparations used for this study have been described in detail previously [ 42]. Briefly, cats were initially anesthetized with Ketamine (10 mg/kg, intramuscular) followed by thiopental sodium (20 mg/kg, intravenous, supplemented as needed during surgery; and at a continuous rate of 1–2 mg/kg/hr, intravenous, during recording). A craniotomy and duratomy were made to introduce recording electrodes into the LGN (anterior: 5.5; lateral 10.5). Animals were paralyzed with Atracurium Besylate (0.6–1 mg/kg/hr, intravenous) to minimize eye movements and artificially ventilated. All surgical and experimental procedures were performed in accordance with United States Department of Agriculture (USDA) guidelines and were approved by the Institutional Animal Care and Use Committee at the State University of New York, State College of Optometry. LGN responses were recorded extracellularly within layer A. Recorded voltage signals were conventionally amplified, filtered, and passed to a computer running the RASPUTIN software package (Plexon, Dallas, Texas, United States). For each cell, spike waveforms were identified initially during the experiment and verified carefully off-line by spike sorting analysis. Cells were classified as X or Y according to their responses to counterphased sine-wave gratings. Only those cells with an SNR > 1 in their responses to repeated presentations of natural scene movies (measured by firing rate in 16-ms bins) were included in the analysis.

### Calculation of BTAs.

BTAs were calculated from simulated and experimental LGN responses as follows [ 9]: first, the spikes in a response that were part of burst events were identified based on the interspike interval criteria described above. Then, the stimulus segments in the window ranging from 384 ms before to 64 ms after the first spike in each burst event were averaged together, yielding a full spatiotemporal BTA. The BTAs of OFF-center cells were reflected about the mean luminance for comparison with the BTAs of ON-center cells. The spatiotemporal BTA was collapsed to a temporal BTA by averaging across all pixels in the RF center and discarding all other pixels. For simulated neurons, the center of the RF was defined as those pixels that were fully within the Gaussian that defined the RF center. For experimentally recorded neurons, the RF center was defined as a 3 × 3 region of pixels (0.6° × 0.6°), centered on the pixel that corresponded to the peak value of the BTA. This value was chosen to match the smallest RF center that is typically observed in cat LGN neurons [ 41].

The BTA as calculated above reflects not only the selectivity in the burst response, but also the temporal correlations inherent in the stimulus. This BTA can be normalized to yield a more accurate characterization of the luminance sequence to which the neuron's burst response is most sensitive by spectral normalization [ 43]. Specifically, the cross-correlation between the natural stimulus and the burst response (which is proportional to the non-normalized BTA) must be normalized by the auto-correlation matrix of the natural stimulus. To perform this normalization, we assumed that the statistics of the movies were stationary and calculated a single temporal auto-correlation matrix for the entire set of movies. However, it should be noted that the normalized BTAs obtained through spectral normalization are only an approximation of the luminance sequence to which the neuron's burst response is most sensitive, because the process of calculating the normalized BTAs assumes a linear relationship between the visual stimulus and burst response which is not strictly valid.

### Simulation of detection tasks.

To simulate the LGN response to different luminance sequences with and without bursts, stimuli were created in which excitatory, inhibitory, or biphasic temporal luminance sequences were superimposed on a background of noise. The temporal scale of the sequences was matched to the observed BTAs of LGN cells. Excitatory sequences were 32 ms in duration, while inhibitory sequences were 128 ms in duration. Biphasic sequences consisted of an inhibitory sequence followed by an excitatory sequence. Both the noise and the sequence were spatially uniform and covered the entire RF. The intensity of the sequence was drawn with equal probability for each trial from the set {0.2,0.3,0.4}. The units of the stimulus were chosen so that the mean firing rate of the IF model at *V _R_* = −65 mV during stimulation with a 16-ms step stimulus was approximately (( *I* − 0.1) × 625) Hz in its linear operating range, where *I* is the intensity of the stimulus. The intensity of the background was randomly chosen from a zero mean, uniform distribution every 16 ms. The width of the noise distribution was varied between 4 and 1/4 times the width of the distribution of sequence intensities, so that the simulation could be run at a variety of stimulus SNRs. LGN responses were simulated using the IFB and IF models with the RF and spike generation parameters described above.

### Analysis of detection tasks.

Simulated responses to different luminance sequences were analyzed using signal detection theory. First the probability distributions of the IF and IFB responses (firing rate in 16-ms bins) under the *S*_1_ and *S*_0_ stimulus conditions were estimated. For all stimuli, condition *S*_1_ corresponded to those 16-ms intervals that followed the excitatory transient of a sequence, and condition *S*_0_ corresponded to all other intervals. Distributions were estimated using trials in which 100 sequences randomly appeared. The time between successive appearances of the sequence was long enough that the effects of responses during the presence of the sequence that fell outside the *S*_1_ interval on the distribution of *S*_0_ responses were minimal.

The detection task involves deciding whether or not a given response *r = R* is due to the appearance of a sequence. The optimal method for detection is that which yields the maximal probability of detection for fixed probability of false alarm. If the response distributions are overlapping, the optimal method is to determine whether the likelihood ratio *R* exceeds some threshold *h* [ 40]. The likelihood ratio is the ratio of probability of observing a given response *r = R* when a sequence has appeared, *p*( *r* = *R* | *S*_1_) to the probability of observing that response at all other times, p( *r* = *R* | *S*_0_). The decision as to whether a given response is due to the appearance of a sequence ( *D*_1_) or due to noise ( *D*_0_) is made as follows:



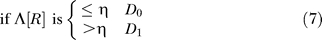



The assumptions underlying this decision criterion are considered in the Discussion.

The reliability of the decision across all possible threshold values can be quantified using an ROC curve. The ROC curve plots the probability of detection *p*( *D*_1_ | *S*_1_) versus the probability of false alarm *p*( *D*_1_ | *S*_0_) for all possible threshold values (see, for example, Figure 3D). The area under the ROC curve is the probability of a correct decision in a two-alternative forced-choice task. At the lowest threshold value, the likelihood ratio at all possible response values lies above the threshold, resulting in certain detection as well as certain false alarm (top right corner of the ROC curve). At the highest threshold value, the likelihood ratio at all possible response values lies below the threshold, meaning no sequence appearances are detected, nor is any noise falsely classified as the appearance of a sequence (bottom left corner of the ROC curve). For the range of threshold values between these extremes, the area under the ROC curve gives a measure of detection performance (specifically, the area under the ROC curve is the probability of a correct decision in a two-alternative, forced-choice task). If the distributions of the response under the two stimulus conditions completely overlap, the ROC curve will lie along the line of equality, resulting in an area of 0.5. If the two distributions are completely separated, the ROC curve will reach the top left corner of the plot, resulting in an area of 1.

## Supporting Information

Video S1 Natural Scene Movie Sample 1 This video segment is an example of the natural scene movies used in this study. The video was recorded by a camera mounted on the head of a freely roaming cat (see Materials and Methods) by members of the laboratory of Peter König at the Institute of Neuroinformatics, ETH/UNI Zürich [ 23]. The white square superimposed on the video indicates the spatial extent of the segments presented in this study.
(573 KB WMV)

Video S2 Natural Scene Movie Sample 2 This video segment is an example of the natural scene movies used in this study. The video was recorded by a camera mounted on the head of a freely roaming cat (see Materials and Methods) by members of the laboratory of Peter König at the Institute of Neuroinformatics, ETH/UNI Zürich [ 23]. The white square superimposed on the video indicates the spatial extent of the segments presented in this study.
(557 KB WMV)

Video S3 Natural Scene Movie Sample 3 This video segment is an example of the natural scene movies used in this study. The video was recorded by a camera mounted on the head of a freely roaming cat (see Materials and Methods) by members of the laboratory of Peter König at the Institute of Neuroinformatics, ETH/UNI Zürich [ 23]. The white square superimposed on the video indicates the spatial extent of the segments presented in this study.
(588 KB WMV)

## Abbreviations

BP: burst percentage
BTA: burst-triggered average
IF: integrate-and-fire
IFB: integrate-and-fire-or-burst
LGN: lateral geniculate nucleus
PIR: post-inhibitory rebound
RF: receptive field
ROC: receiver operating characteristic
SNR: signal-to-noise ratio
